# Quantification of Somatic Chromosomal Rearrangements in Circulating Cell-Free DNA from Ovarian Cancers

**DOI:** 10.1038/srep29831

**Published:** 2016-07-20

**Authors:** Faye R. Harris, Irina V. Kovtun, James Smadbeck, Francesco Multinu, Aminah Jatoi, Farhad Kosari, Kimberly R. Kalli, Stephen J. Murphy, Geoffrey C. Halling, Sarah H. Johnson, Minetta C. Liu, Andrea Mariani, George Vasmatzis

**Affiliations:** 1 Department of Molecular Medicine, Mayo Clinic, Rochester, MN 55905, USA; 2 Department of Molecular Pharmacology and Experimental Therapeutics, Mayo Clinic, Rochester, MN 55905, USA; 3 Gynecology, Mayo Clinic, Rochester, MN 55905, USA; 4 Medical Oncology, Mayo Clinic, Rochester, MN 55905 USA

## Abstract

Recently, the use of a liquid biopsy has shown promise in monitoring tumor burden. While point mutations have been extensively studied, chromosomal rearrangements have demonstrated greater tumor specificity. Such rearrangements can be identified in the tumor and subsequently detected in the plasma of patients using quantitative PCR (qPCR). In this study we used a whole-genome mate-pair protocol to characterize a landscape of genomic rearrangements in the primary tumors of ten ovarian cancer patients. Individualized tumor-specific primer panels of aberrant chromosomal junctions were identified for each case and detected by qPCR within the cell-free DNA. Selected chromosomal junctions were detected in pre-surgically drawn blood in eight of the ten patients. Of these eight, three demonstrated the continued presence of circulating tumor DNA (ctDNA) post-surgery, consistent with their documented presence of disease, and in five ctDNA was undetectable in the post-surgical blood collection, consistent with their lack of detectable disease. The ctDNA fraction was calculated using a novel algorithm designed for the unique challenges of quantifying ctDNA using qPCR to allow observations of real-time tumor dynamics. In summary, a panel of individualized junctions derived from tumor DNA could be an effective way to monitor cancer patients for relapse and therapeutic efficacy using cfDNA.

The analysis of circulating cell-free DNA (cfDNA) shows exciting promise for the detection of genomic alterations associated with cancer. CfDNA is a natural phenomenon and is thought to originate from DNA released into the circulation from apoptotic cells deriving primarily from normal noncancerous tissue. However, in cancer patients the release of DNA from necrotic tumor cells (ctDNA) constitutes a significant portion of total cfDNA^1^.

Strong correlations between ctDNA and disease prognosis have been reported in advanced colorectal cancer^2^. Additionally, ctDNA levels were demonstrated to correlate with progression or remission in breast^3^ and prostate^4^ cancers, and in melanoma^5^. Patients with advanced stage cancers have been reported to have higher levels of ctDNA than earlier stage patients across several different cancer types^6^, with specificity nearing 100%^7^. Encouragingly, serial monitoring of ctDNA in breast cancer was suggested to be more informative than standard techniques used to detect recurrence clinically: In a report by Olsen and colleagues, ctDNA was observed an average of 11 months before metastases were detected clinically in 86% of patients, and was undetectable in those without recurrence^8^. The predicted short half-life of ctDNA of about two hours^2^ allows a real-time glimpse into tumor dynamics, enhancing its immediacy in monitoring therapeutic efficacy. Thus, the detection of ctDNA has great potential as a specific biomarker for monitoring tumor burden.

Ovarian Cancer (OC) is one of the most common cancer deaths among patients with gynecologic malignancies, with approximately 21,290 new cases diagnosed and 14,000 deaths estimated for 2015^9^. Most OC patients are diagnosed with late-stage invasive disease, and although the majority experience initial remission after surgical debulking and adjuvant chemotherapy, about 75% relapse and develop chemo-resistant disease^10^. While measurement of blood levels of the CA-125 protein has been a widely used biomarker for OC for over two decades, this circulating protein is neither sensitive nor specific^11^. Moreover, other biomarkers such as HE4 that have been recently proposed need further investigation^12^. Thus, there is a need for additional biomarkers, both for screening and monitoring OC, that could complement and improve upon CA-125 and other available biomarkers.

Previously ctDNA studies in OC have focused on the identification of point mutations in TP53 13, a gene panel consisting of known tumor drivers^14^, whole exome^15^,^16^ or paired-end DNA direct sequencing of cfDNA^6^. In one study, a fusion gene involving FGFR2 was identified in an OC case^17^. Serial blood collections were tested for the presence of the fusion in ctDNA over the course of multiple treatments and found the detection of the FGFR2 fusion product to be a more sensitive biomarker for tumor recurrence than CA-125 17. Recently, ctDNA was detected an average of 7 months preceding positive CT scans for recurrence in 44 patients with a range of gynecological cancers, including 22 with ovarian cancer^18^. Collectively, these studies provide evidence supporting the feasibility of disease monitoring using ctDNA in OC. Reported quantification methods differ between studies, however, making it difficult to adapt a ctDNA detection approach for clinical use.

We report here an individualized, sensitive and specific approach for disease surveillance and therapeutic response monitoring in OC. A next-generation sequencing mate-pair protocol (MPseq) was used to identify somatic structural genomic alterations in primary tumors and a subset were quantitated in blood samples of each patient. Unique for each tumor, these aberrant DNA junctions involved genes with protein products that could be therapeutically targetable or were characteristic of significant clonal populations. A novel algorithm utilizing qPCR values for tumor-specific junctions and a housekeeping gene was applied to quantify the relative level of mutant DNA fragments to assess tumor burden. Thus, this proof-of-concept study describes a methodology that could serve as a useful tool to monitor disease regression or recurrence in a novel quantitative manner.

## Results

### Landscape of structural rearrangements in serous ovarian carcinoma

**Figure 1:**
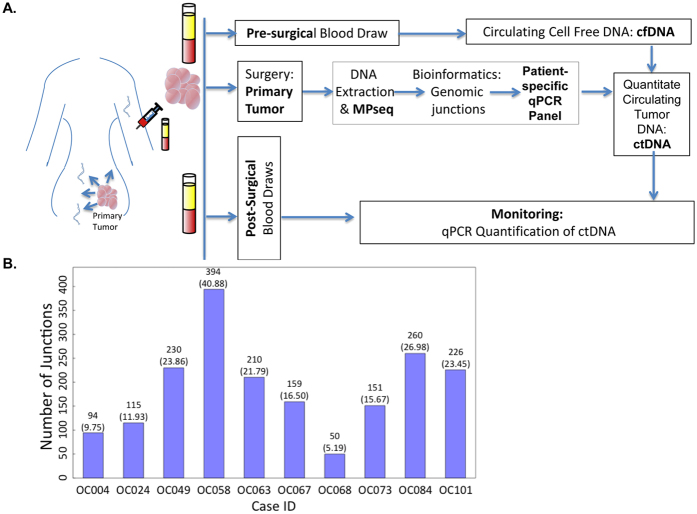
Assay Schema. (**A**) Blood is drawn before and after surgery. DNA from tumor is sequenced using the next-generation mate-pair sequencing (MPseq) protocol to identify chromosomal rearrangements. Several junctions are chosen to construct a personalized panel for each tumor. Percent of ctDNA out of total cfDNA is calculated at each time point of blood collection. (**B**) Number of junctions identified in cohort of 10 cases of serous stage 3 ovarian cancer. Count numbers are above the bars with the expected false negative count in parenthesis. cfDNA: cell-free DNA; ctDNA: circulating tumor DNA.

Figure 1A exemplifies the workflow process utilized in this study for monitoring tumor-defined rearrangements in the blood. Ten patients with high-grade serous OC stage III-IV (Table 1) were selected retrospectively with plasma samples available from both pre- and post-operative surgery blood draws. DNA was initially isolated from fresh-frozen surgically resected primary tumor tissue and whole-genome sequenced using the MPseq protocol^19^,^20^. Structural DNA variant analysis was performed on the 10 patients using previously published bioinformatics algorithms developed in our group^19^,^20^.

Large genomic rearrangements were highly abundant in all cases analyzed (Fig. 1B). An average of 188 junctions/case (median 184) were observed between the 10 cases, with a range of 50–389 (Fig. 1B) when counting junctions with at least 3 supporting mate-pairs. Additional junctions expected based on the portion of the reference genome that is unmapped range from 5.19–40.88 (Fig. 1B).

**Figure 2:**
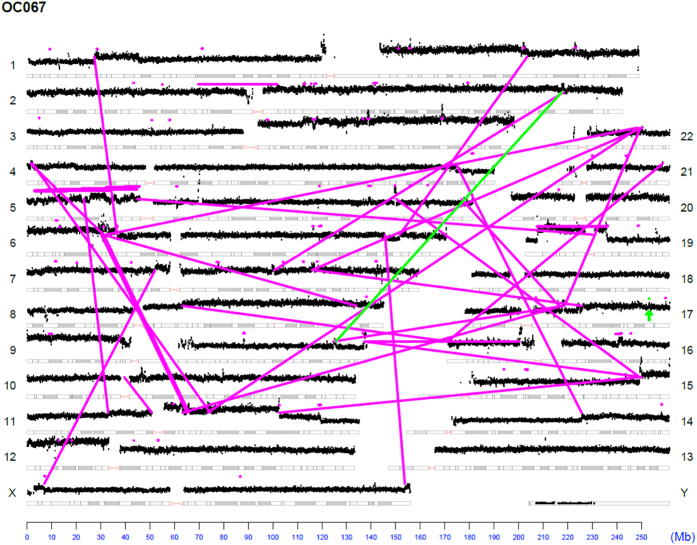
Next Generation mate-pair sequencing of primary Ovarian Cancer. (**A**) Genome plot of exemplar case OC067. Chromosomes are listed on the left and right Y-axis’; basepair position is on the X axis. Grey cytobands indicate genomic loci bands. The height of the black dots each represents the average number of reads over 30 k bases. Magenta dots indicate small intrachromosomal rearrangements, while the magenta lines indicate interchromosomal rearrangements or larger intrachromosomal junctions. Green lines/dots indicate the junctions of interest monitored in the blood.

The landscape of genomic rearrangements for representative sample OC067 is presented as a genome plot (Fig. 2). The number and span of alterations observed in sample OC067 are typical of serous ovarian patients. The genome plot details chromosomal rearrangements of the genome. Inter-chromosomal junctions appear as magenta lines to illustrate the relative locations of the breakpoints. Intra-chromosomal junctions appear as magenta horizontal lines over the affected area or magenta dots for small focal alterations. Junctions further investigated are shown in green. Genome plots for additional cases are available in the supplemental materials (Supplemental Figures S1–S8).

### Quantification of cell free DNA

Pre- and post-surgical plasma DNA was processed for each case using the Qiagen circulating nucleic acid kit. CfDNA was successfully detected in all samples. Concentration ranged from 9 to 35 ng/ml (Supplemental Figure 9A). When compared, the pre-surgical plasma samples showed a slightly higher quantity of cfDNA (median 16.5 ng/ml), than post-surgical plasma samples (median 13.2 ng/ml), but not at a statistically significant level (Supplemental Figure 9B).

### PCR screening of junctions in cfDNA

**Figure 3:**
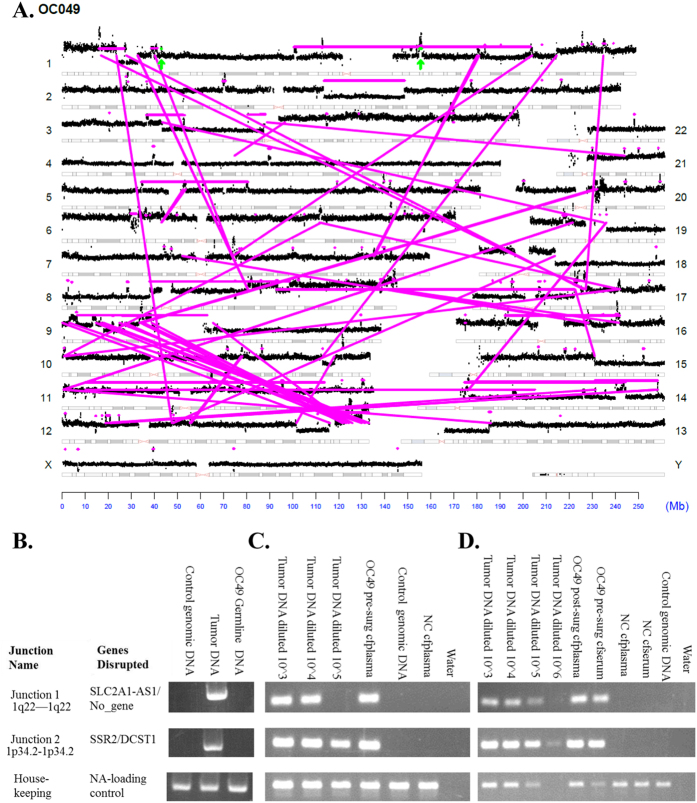
Detection of selected junctions in blood. (**A**) Genome plot of case OC049, interpreted as in Fig. 2. (**B**) PCR validation of two selected junctions (as indicated) in tumor tissue of case OC049. (**C**,**D**) Agarose gels of qPCR showing junctions 1 and 2 of OC049 in pre-surgical plasma (**C**), pre-surgical serum, and post-surgical plasma (**D**).

From rearrangements detected in each case, panels of PCR primers spanning the detected breakpoints were designed as unique tumor markers. In representative exemplar case OC049 a total of 229 chromosomal junctions were observed in the primary tumor (Figs 1B and 3A). The two events chosen for ctDNA analysis, highlighted in green, both predict copy number gains from intra-chromosomal rearrangements on chromosome 1. Each junction was initially verified in the primary tumor DNA and demonstrated to be a true somatic event absent from the germline (Fig. 3B). Subsequent qPCR screening of pre- and post-surgical plasma cfDNA of case OC049 showed amplification products (Fig. 3C,D) and Sybr Green melting curves, which were identical to those seen in the primary tumor and the identity of the products was confirmed by Sanger sequencing (data not shown).

For eight out of the ten patients tested, junctions identified in DNA of the primary tumor were also detected in the cfDNA derived from pre-surgical plasma (Table 1). Of these eight, three demonstrated a continued presence of the ctDNA post-surgery, consistent with the presence of disease, which was documented at the time of the blood draw. In five patients, ctDNA could not be detected post-surgery, in accord with the lack of detectable disease observed at the time of the blood draw. Thus, for these eight cases, perfect concordance was observed between ctDNA detection and clinical presentation at the time of the blood draw. In two of the ten cases, somatic rearrangements could not be detected in the patient plasma.

### Quantitative PCR measurement of ctDNA breakpoints in cfDNA

**Figure 4:**
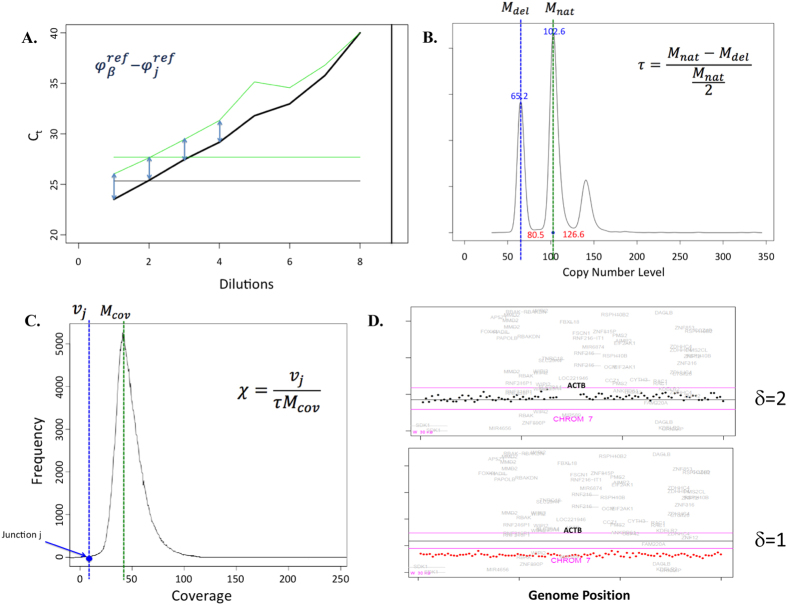
Quantification of ctDNA fraction. (**A**) Plot showing reference values for Ct (derived from qPCR) for housekeeping gene and junction of interest, calculated from a standard curve constructed using the Ct’s for four dilutions of DNA of corresponding tumor sample (**B**) Plot showing distribution of mate-pair reads corresponding to copy number variants in the tumor sample. Fraction of tumor was calculated using copy number probability density function. The largest peak corresponds to diploid copy number level (M_nat_), the lower peak corresponds to a single copy deletion (M_del_) and the third peak to gains. (**C**) Plot showing distribution of mate-pair reads as a function of coverage. The fraction of cells in the tumor harboring a specific junction was calculated using the expected coverage (M_cov_), the tumor fraction (τ), and the number of mate-pairs associated with a junction (ν_j_). (**D**) Count plot showing copy number changes (δ) at the locus of housekeeping gene ACTB with 2 examples showing wild-type 2 copy state (top) and copy loss (bottom).

Quantitative PCR assays were developed and used to quantitate the amount of each aberrant chromosomal junction relative to the control beta actin housekeeping gene (Fig. 4). Primers unique to each junction were designed and optimized for qPCR. The difference in primer efficiencies was corrected by normalizing standard qPCR curves of tumor DNA for each individual case. 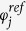 and 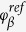 were determined by taking the mean of the Ct’s of a junction and beta actin (respectively) for the first four highest concentrations of the tumor DNA (as they show the best linearity) (Fig. 4A). The standard curves were further corrected to take into account: 1) the percent of normal cells in the sequenced tumor DNA, 2) intra-tumoral heterogeneity represented by unique clones within the tumor, and 3) CNVs for the housekeeping gene in the tumor.

The fraction of tumor cells in the tumor tissue, *τ*, was subsequently calculated using the tumor fraction equation (Fig. 4B). By determining the mode of MPseq read depth (M), we calculated the coverage for both the normal 2-copy state (M_nat_) and the deleted, 1-copy state (M_del_). Example reads for case OC049 are plotted according to their frequency; with resulting peaks correlating with copy number status (Fig. 4B). When M_nat_ equals 100, this corresponds to a diploid state, while sequences associated with a loss of one copy (M_del_) center around value of 50 (Fig. 4B). Conversely, the peak centered at 150, corresponds to a single gain. The difference between the value of the M_del_ peak and the value of 50 on the X-axis represents the fraction of normal, non-tumor DNA in the sample. To correct for intra-tumoral heterogeneity, *χ* was calculated using the tumor heterogeneity equation (Fig. 4C). It represents the fraction of the clone harboring a specific junction within the tumor. Finally, the value δ is used to adjust for any CNV of beta actin (β_T_) in the tumor: δ = 1 if β_T_ loses one copy, δ = 2 if β_T_ is normal, and δ = 3 corresponds to one copy gain in the effective correction equation (Fig. 4D).

The final Quantitative Circulating Tumor Percentage Equation (Equation 1) was derived for calculating the percentage of ctDNA present in cfDNA, with the more in-depth derivation presented in materials and methods.
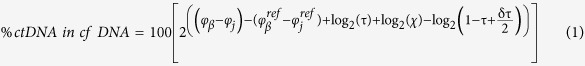


**Figure 5:**
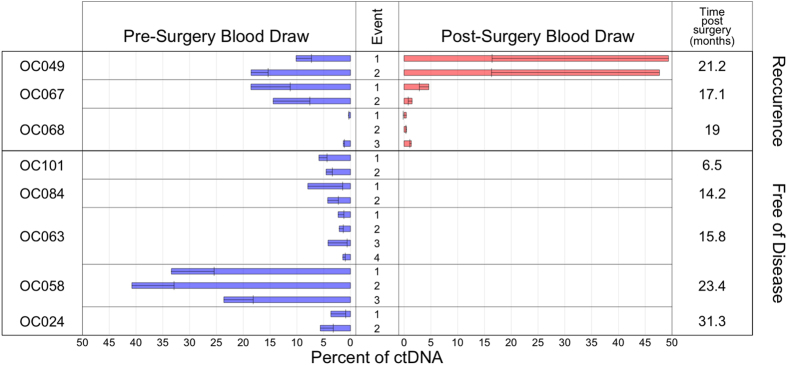
Percent ctDNA. Histogram showing the calculated percent (x-axis) of ctDNA in cfDNA for each detected rearrangement of each case. Error bars: calculated standard deviation.

The Quantitative Circulating Tumor Percentage Equation was used to estimate ctDNA in all cases (Table 1, Fig. 5). For case OC049, the pre-surgical blood sample was found to contain 10.1% and 18.6% ctDNA for the screened junctions, respectively. In the post-surgical blood sample, ctDNA was 49.3% and 47.6% (Fig. 5), consistent with the presence of disease 21 months post-surgery, thus suggesting a poor clinical prognosis. This result correlated with the recurrence of the cancer documented in the clinical record of the patient, who died of disease 15 days following the second blood draw.

Qualitatively, our results matched clinical record data (when available) for the presence of disease at each draw. There was also a strong relationship between post-surgical CA-125 measurement and post-surgical qualitative qPCR results in our patients for whom comparisons could be made (Table 1). Post-surgical cases with low CA-125 (<30 U/mL) coincided with an absence of detectable ctDNA. We found that the percentage of ctDNA varied highly amongst the 8 patients analyzed, with values ranging from 0.3% to 49.3%. Standard deviations within each individualized panel of junctions tested for each case ranged from 0.0–32.9 %ctDNA (mean 3.3; median 0.6), showing a variability that can be attributed to heterogeneity within the tumor and/or differences in the amount of DNA released from corresponding clone.

## Discussion

We report here a new algorithm, based on the quantification of specific somatic junctions in the blood of OC patients, to assess the fraction of ctDNA. Large numbers of chromosomal rearrangements were identified in the primary tumors of ten OC cases using a MPseq protocol. Individual somatic junctions were detected in the blood of eight (80%) patients (Table 1). The relative amount of tumor-specific fragments varied between cases and correlated with disease status with the exception of two cases where ctDNA was undetectable, even in pre-surgical blood.

Quantification methods for the ctDNA fraction of cfDNA reported up to date vary tremendously, making cross-comparisons difficult. In one study, ctDNA was quantified using standard curves constructed from serial dilutions of the purified rearrangement product^17^. Although this approach provides a more accurate assessment of ctDNA quantity, it would be prohibitively expensive and time consuming in a high through-put environment. The most commonly used methods involve calculation of delta Ct’s derived from qPCR, or digital PCR results. This approach, however, often neglects to factor in several important variables which impact final results. The qPCR algorithm we present in this study takes into account multiple parameters which affect accurate assessment of ctDNA quantities and is relatively inexpensive.

The detection of chromosomal junctions in ctDNA offers several benefits over the detection of point mutations. First, it is extremely specific and robust, and performance is not affected by the abundance of fragments from normal DNA^21^,^22^,^23^,^24^. Second, it is more sensitive than the detection of somatic mutations, which are often at the threshold of sequencing error (~0.01%)^7^. Indeed, comparing the detection sensitivity of junctions to point mutations across multiple solid tumor types revealed greater sensitivity for the former^6^. Third, rearrangements leading to gene fusions can result in potentially clinically targetable protein products, the detection of which is fortuitously aided by increased copy numbers, whereas point mutations are typically present as one genome copy^6^. There are, however, several weaknesses to our approach: MPseq requires fresh frozen tumor or biopsy tissue which may not be routinely available. It is also a little more laborious since no common rearrangements are observed in OC, so each case demands construction of an individual panel for detection. Because alterations need to be initially identified in the tumor, and then tracked in the blood, our method could not be applied in an OC screening application.

The recent explosion of ctDNA studies and the rapid increase in sensitivity and specificity of methodologies for ctDNA detection allude to a coming clinical integration, and point to the possibility of routine measurement of ctDNA in cancer patients in the near future. OC patients appear particularly suited to benefit from this approach, as they shed relatively higher amounts of ctDNA than those with other cancers^6^ and are also at great risk of recurrence following initial chemotherapy.

We envision performing sequencing of the tumor and creating a personalized monitoring panel after the commencement of treatment. Additionally, this may be the ideal time to identify potential therapeutic targets unique to the cancer. Thus, should a patient recur, as most patients with ovarian cancer will^10^, a personalized treatment plan accompanied by a customized monitoring assay can be ready. By monitoring fluctuations of ctDNA% as a proxy for tumor burden, clinicians can infer tumor progression and response to treatment. Patients with detectable ctDNA post-surgery may benefit from closer follow-up. If the tumor fails to respond to a given drug, it can be discontinued –saving the patient unnecessary side-effects and costs. Fusion targets of therapy could likewise be monitored for response. Conceivably junctions indicative of specific clonal populations within the tumor DNA may be monitored for differential responses to treatment. Furthermore, early identification of nonresponders to conventional therapy may help physicians to select alternative treatments and identify patient subgroups eligible for clinical trials.

Recently, ctDNA increases in breast cancer were found to indicate recurrence earlier than rises in CTCs or CA 15-3. Furthermore, detection of ctDNA in half of these cases was possible an average of 5 months before radiologically confirmed disease progression was observed^3^,^25^. Similar results have been reported in gynecological cancer^18^. More work will need to be done in OC patients to study how fluctuations in ctDNA% correlate with other clinical indicators of recurrence over multiple time-points.

Our novel algorithm helps to bring a liquid biopsy protocol closer to routine use in clinical practice. Following acquisition of tumor tissue via surgery or biopsy, the generation of individualized junction signature panels could be designed within three weeks. Our assay would provide clinicians with individualized tumor response information that could be used with great flexibility in case management.

## Materials and Methods

### Case Selection and Sample Processing

Cases were selected out of 100 consecutive patients with suspected epithelial serous stage IIIC-IV OC seen at the Mayo Clinic in Rochester, MN from 8/1/2010–4/30/2012 with available fresh frozen primary tumor and both pre- and post-surgical blood. Post-surgical draws were chosen as available for this retrospective study. Late stage OC patients were selected because of their high tumor burden thus greater ctDNA content^6^ and in order to align the study with the most common clinical presentation^18^. The study was approved by Mayo Clinic’s Institutional Review Board (IRB). All experiments were performed in accordance with the approved guidelines of the Mayo Clinic IRB. Each participant provided written informed consent for use of their biospecimens and clinical data in research. A total of 10 patients’ primary tumors were assessed for tumor purity by a pathologist for at least 60% tumor and macrodissected to enrich for tumor content. DNA was isolated using the Gentra Puregene Blood kit (Qiagen, MD, USA; 158445). DNA was sequenced using the MPseq protocol and analyzed for structural variations. CA-125 was measured within 20 days of the associated cfDNA blood draw. CfDNA was isolated from 3 mL of double-spun plasma using the Circulating Nucleic Acid kit (Qiagen, MD, USA; 55114). Quantification of total cfDNA was performed by the Quant-iT PicoGreen dsDNA reagent (Invitrogen, Eugene, OR; P7581) (Supplemental Figure S9). Three milliliters of plasma or serum from individuals without cancer were pooled and processed in the same way for a negative control (NC cfplasma/NC cfserum).

### Next-Generation Sequencing and Validation of Genomic Rearrangements

DNA from primary tumor was prepared using the Illumina MPseq Kit following the manufacturer’s instructions and sequenced as two libraries per lane on the Illumina HiSeq2000 platform^19^. The sequencing data was mapped using the previously described binary indexing mapping algorithm (BIMA)^20^; post-mapping processing was performed by SVAtools, a suite of R scripts also developed in our group. Bridged coverage and fragment size information can be found in Supplemental Figure S10. The read-to-reference-genome algorithm concurrently mapped both 100 bp MP reads across the whole human genome (hg20/GRCh38). SVAtools first removes replicate fragments and then uses a greedy custom clustering algorithm and a system of masks and filters to detect interchromosomal rearrangements or intrachromosomal rearrangements ≥45 kb. Concordantly mapping MP reads were used to determine copy number across the genome. A minimum cluster of three discordant mapping MP reads was required to identify a chromosomal rearrangement (Fig. 1B)^19^,^20^. Chromosomal rearrangements were verified in patient tumor DNA samples as described previously^19^. Pooled human genomic DNA (gDNA) was used as a control (Promega, Madison, WI; G304A). DNA bands were Sanger sequenced to confirm rearrangements and identify precise genomic break points.

### Generation of Individualized Monitoring Panels

A panel of 2 to 4 unique junctions was selected from primary MPseq data for each case to be used for detection in the blood (Supplemental Table S1). Selection prioritized junctions indicative of gene alterations that had the potential for therapeutic intervention and/or were supported by at least 15 associated mate-pairs. Potentially targetable alterations were identified using the PANDA (Pathway and Annotation) tool, which allows visualization of data in the context of pathways and includes annotations of drug-gene and gene-disease interactions (http://bioinformaticstools.mayo.edu/Panda/). The results were then cross-referenced with a database, which includes drugs approved for clinical use (https://clinicaltrials.gov/). After identifying the breakpoint for each junction of interest, new primers were designed to generate products <150 bps to be more suitable for qPCR and account for the fragmentation of ctDNA^26^. Junctions were confirmed as specific for somatic rearrangements by PCR using DNA from the pooled plasma of normal controls and buffy coat DNA for each patient. GAPDH amplification was used as an internal control (forward primer: GAACGGGAAGCTCACTGGCATG; reverse primer: CTAGACGGCAGGTCAGGTCCACC).

### Detection of ctDNA and Analytical Methods

Detection of ctDNA was accomplished using qPCR, performed as follows: 150 nm of primers were added to 2x Sybr Green (Invitrogen, Eugene, OR; 4367659) and 3–4 ul of template DNA. Serial dilutions of patient tumor DNA were used as positive controls and to allow quantification of the mutant product. Negative controls included NCcfDNA (normal control cfDNA), pooled gDNA, and water as a No Template Control (NTC). Beta actin was amplified in each qPCR for normalization purposes (forward primer: TCCTCTCCCAAGTCCACACA; reverse primer: GCACGAAGGCTCATCATTCA). qPCR was run on Applied Biosystems 7900 HT using a standard protocol (40 cycles of 95 °C 15 seconds and 60 °C 1 minute). Products were resolved by 4% agarose gel electrophoresis for visualization purposes.

### Derivation of equations for quantification of ctDNA

#### Percentage of ctDNA in cfDNA


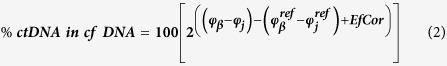


The above formula presents the derivation of the final formula for quantitation of each aberrant tumor chromosomal junction relative to control housekeeping gene. Where *φ*_*j*_ and *φ*_*β*_ are defined as the Ct values for individual tumor-specific junctions and for the housekeeping gene beta actin, respectively. The middle derivation additionally corrects for the difference in primer efficiencies by normalizing standard qPCR curves of tumor DNA for each individual case: where, 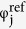
and 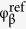 are determined by taking the mean of the Ct’s of a junction and beta actin (respectively) for the first four highest concentrations of the tumor DNA. Each qPCR reaction was run in duplicate, which was used to determine a standard deviation for each Ct value. These standard deviations were used to calculate an overall standard deviation for each ctDNA (Fig. 5).

The final derivation incorporated the EfCor variable correction for confounding factors of the percentage of normal cells, intratumeral heterogeneity and CNV of the house keeping genes:
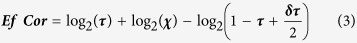


The initial derivation incorporates the tumor fraction and heterogeneity equations (Equations 4 and 5).

#### Tumor fraction equation

Fraction of tumor cells in tumor tissue (*τ*):
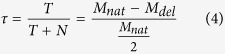
where, T is tumor DNA (corresponding to a single allele), N is normal 2-copy state DNA, M_nat_ and M_del_ are coverage for the normal 2-copy state and the deleted, 1-copy state, respectively.

#### Heterogeneity equation

Correction for intra-tumoral heterogeneity (*χ*):
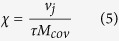


where, ν_j_ is the number of associated mate-pairs for each junction, *τ* is the fraction of tumor DNA in the tissue sample calculated in tumor fraction equation (equation 4), and M_cov_ is the mode of the bridge coverage for a single allele.

The final derivation of the effective correlation equation (Equation 3) uses the value δ to adjust for any CNV of beta actin (β_T_) in the tumor: δ = 1 if β_T_ loses one copy, δ = 2 if β_T_ is normal, and δ = 3 corresponds to one copy gain.

## Additional Information

**How to cite this article**: Harris, F. R. *et al*. Quantification of Somatic Chromosomal Rearrangements in Circulating Cell-Free DNA from Ovarian Cancers. *Sci. Rep.* **6**, 29831; doi: 10.1038/srep29831 (2016).

## Supplementary Material

Supplementary Information

## Figures and Tables

**Table 1:** Patients’ clinical characteristics.

Case ID	Stage	CA-125 pre-surgery (U/mL)	Junctions detected at pre-surgery	Time from surgery to post-surgical blood collection (months)	CA-125 at post-surgical blood collection (U/mL)	Junctions detected at post-surgical blood collection	Recurrence present at post-surgical blood collection	Vital Status	Total follow-up from surgery (months)
OC004	4	8043	No	35.9	NT	No	Yes*	DOD	52.9
OC024	3C	611	Yes	31.3	10	No	No	NED	52.3
OC049	3C	1771	Yes	21.2	2840	Yes	Yes*	DOD	21.7
OC058	3C	152	Yes	23.4	NT	No	No	NED	37.7
OC063	3C	844	Yes	15.8	NT	No	Unknown	Alive (disease unknown)	15.8
OC067	3C	1233	Yes	17.1	1500	Yes	Yes*	DOD	33.7
OC068	3C	7041	Yes	19	3337	Yes	Yes*	AWD	44.0
OC073	4	2435	No	20.5	54**	No	No	AWD	44.5
OC084	3C	1330	Yes	14.2	30	No	No	AWD	40.2
OC101	3C	3430	Yes	6.5	13	No	No	NED	30.5

Correlation of the detection of junctions in blood with clinical recurrence. NT-not tested; DOD-dead of disease; NED- no evidence of disease; AWD- alive with disease.

*Clinically evident symptomatic disease at imaging.

**Tested 1.5 months before post-surgical blood collection.
