# Supplementary material for: Quantification of Somatic Chromosomal Rearrangements in Circulating Cell-Free DNA from Ovarian Cancers
Source: Sci Rep. 2016 Jul 20;6:29831. doi: 10.1038/srep29831 (PMC4951715; doi:10.1038/srep29831)
Supplement: Supplementary Information [file srep29831-s1.pdf]

Supplemental Information

Quantification of Somatic Chromosomal Rearrangements in Circulating Cell-Free DNA from Ovarian Cancers

Faye R. Harris[1]**, Irina V. Kovtun[2]**, James Smadbeck[1], Francesco Multinu[3], Aminah Jatoi[4], Farhad Kosari[1], Kimberly R. Kalli[4], Stephen J. Murphy[1], Geoffrey C. Halling[1], Sarah H. Johnson[1], Minetta C. Liu[4], Andrea Mariani[3]*, George Vasmatzis[1]*

* *These authors contributed equally to the study

* Corresponding authors

Supplemental
Figure S1

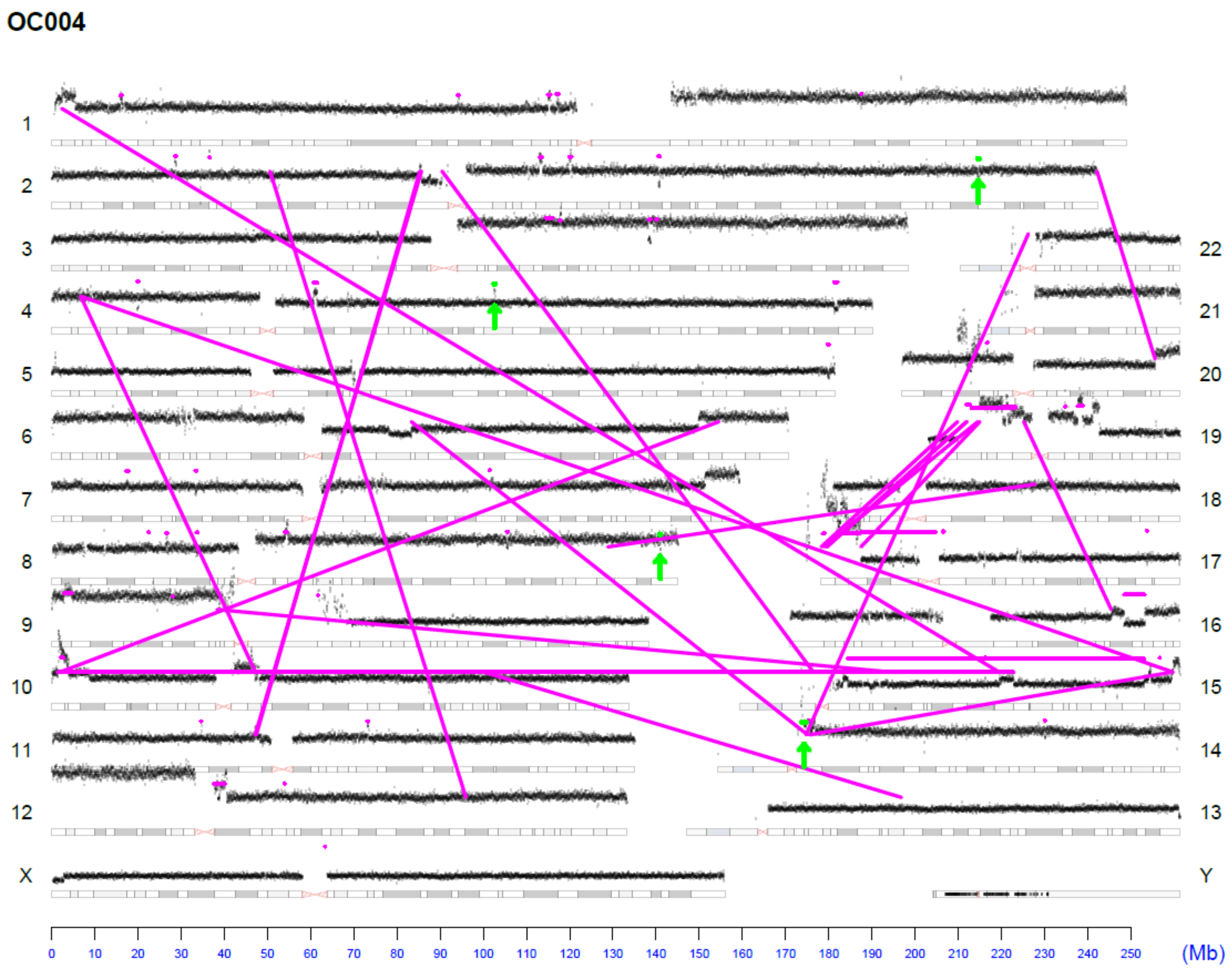

Supplemental
Figure S2

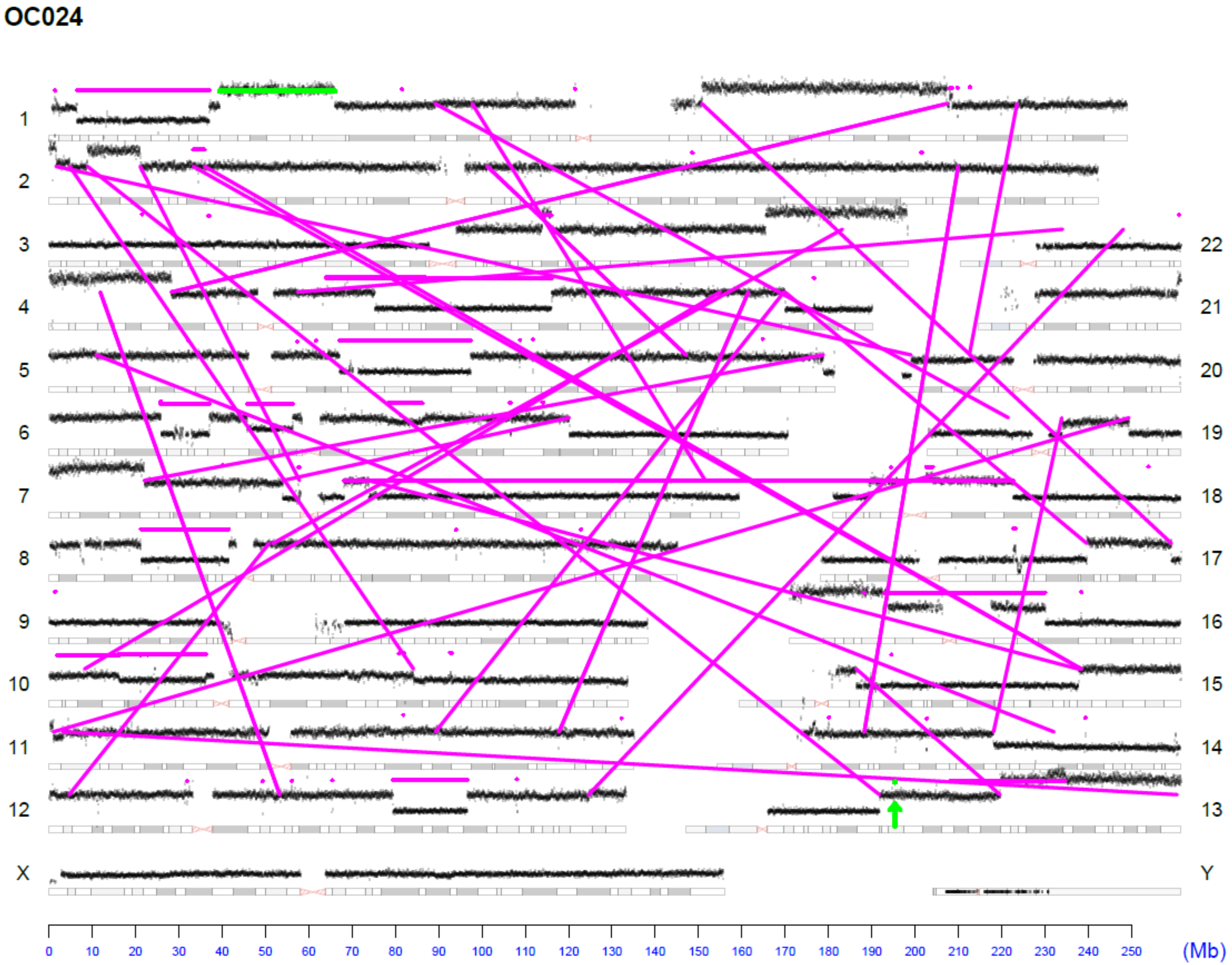

Supplemental
Figure S3

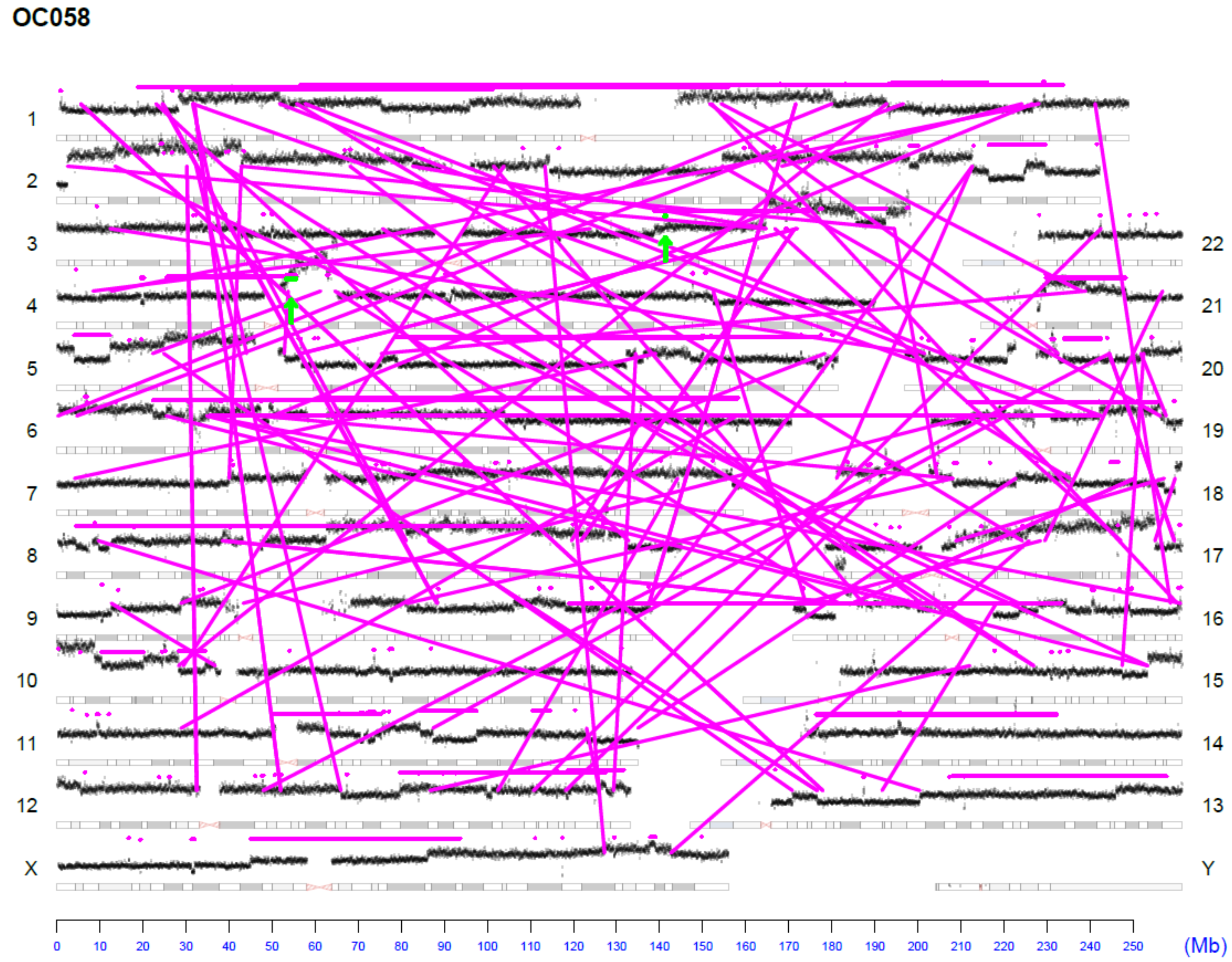

Supplemental
Figure S4

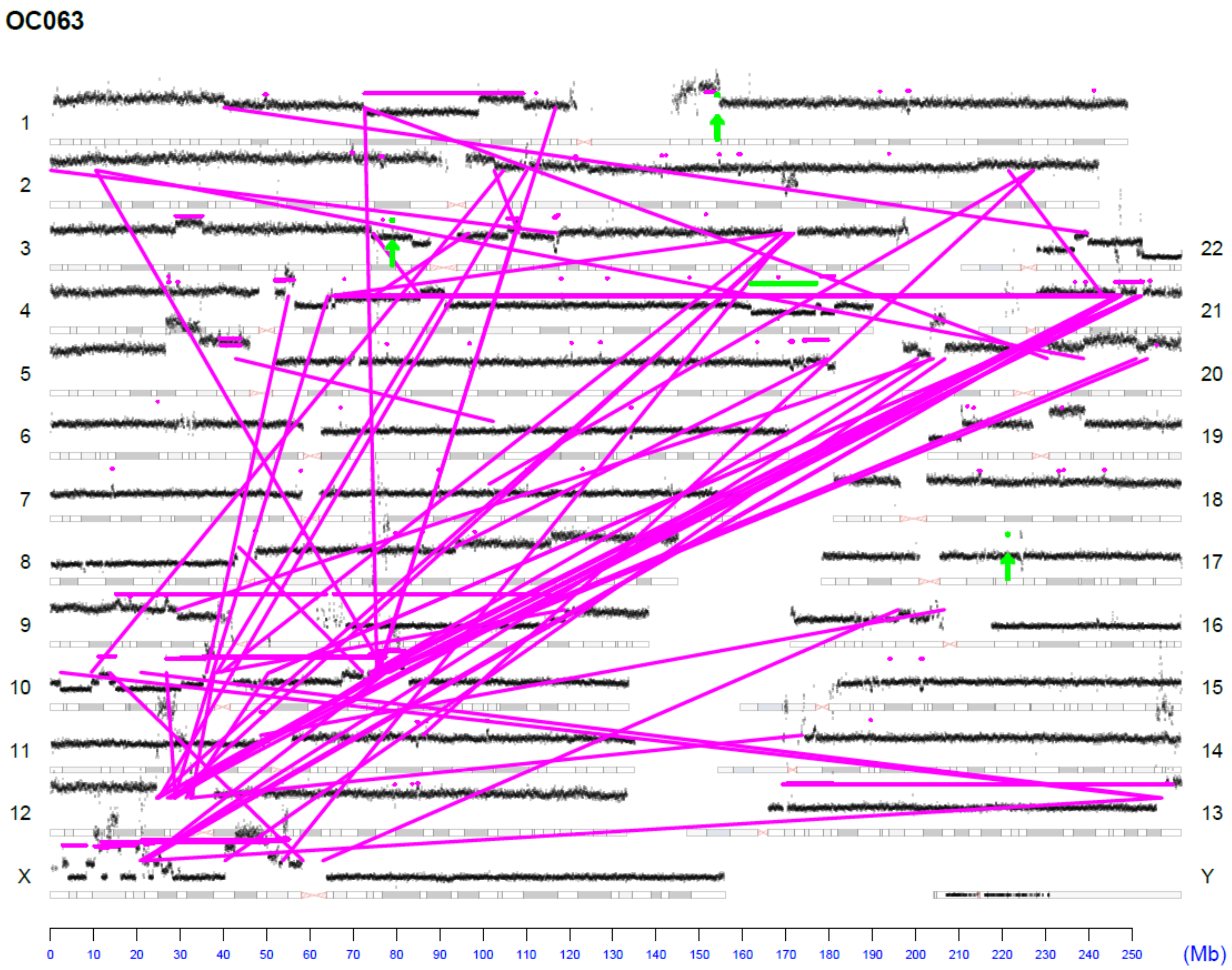

Supplemental
Figure S5

OC068

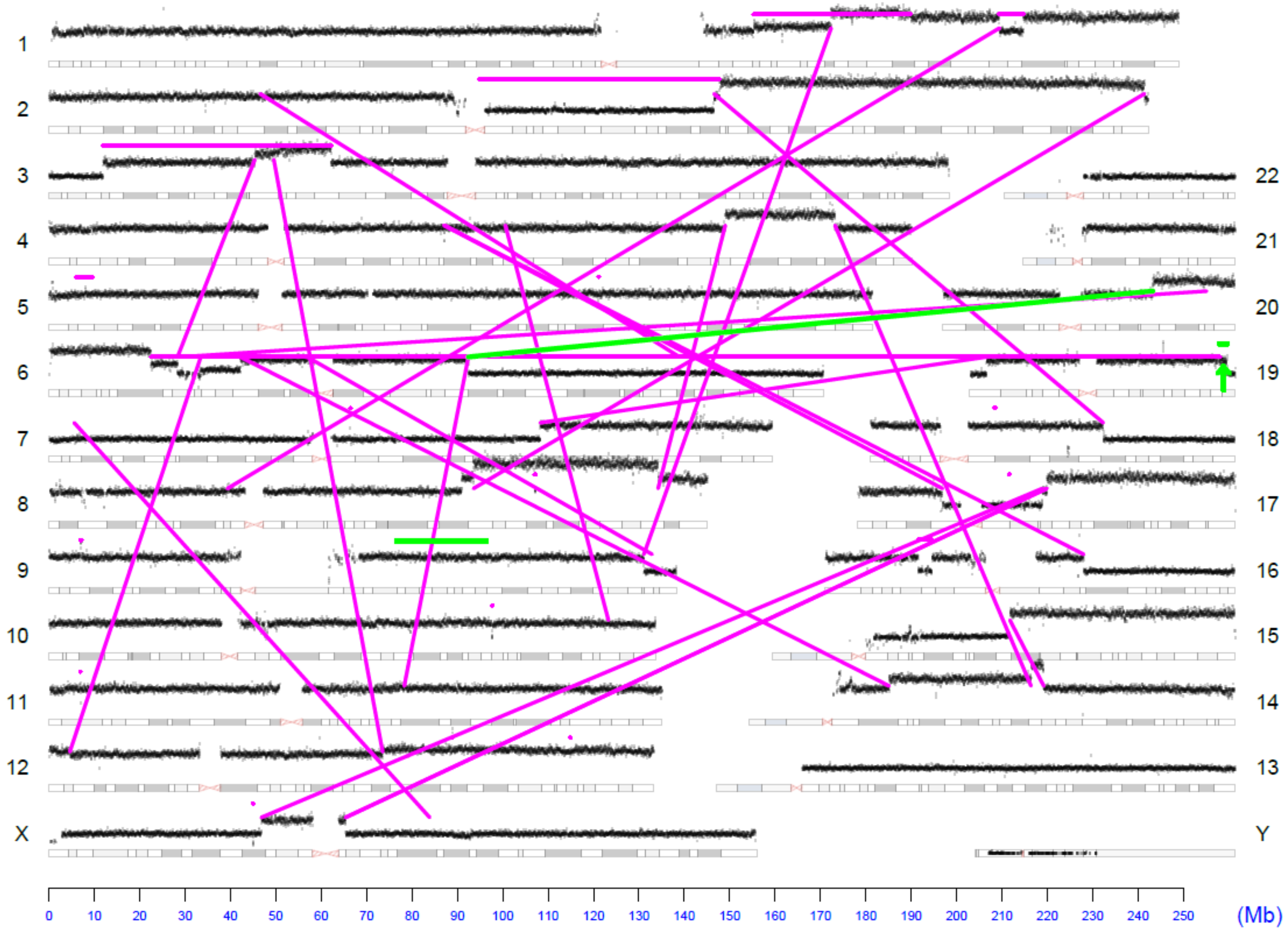

Supplemental
Figure S6

OC073

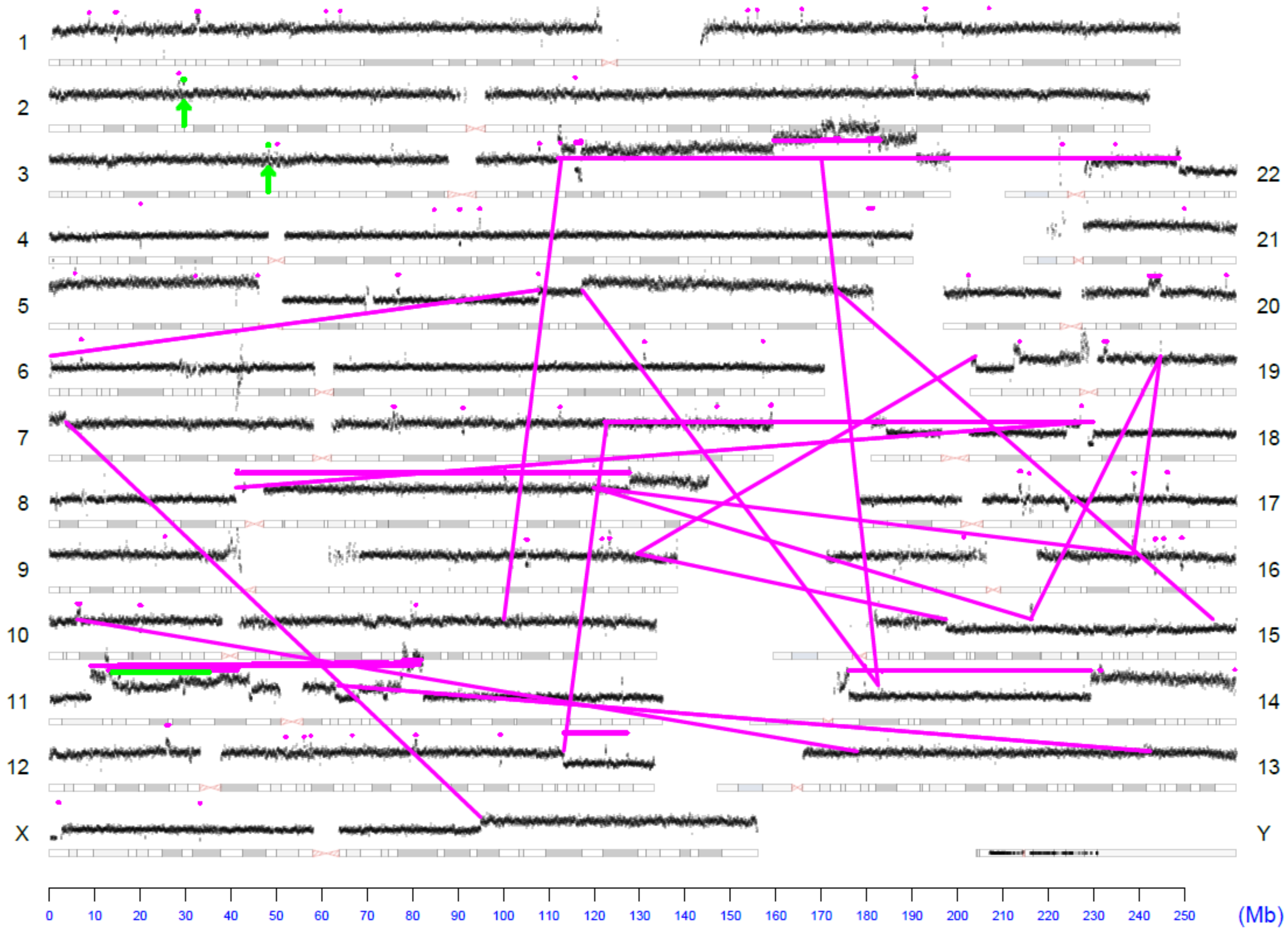

Supplemental
Figure S7

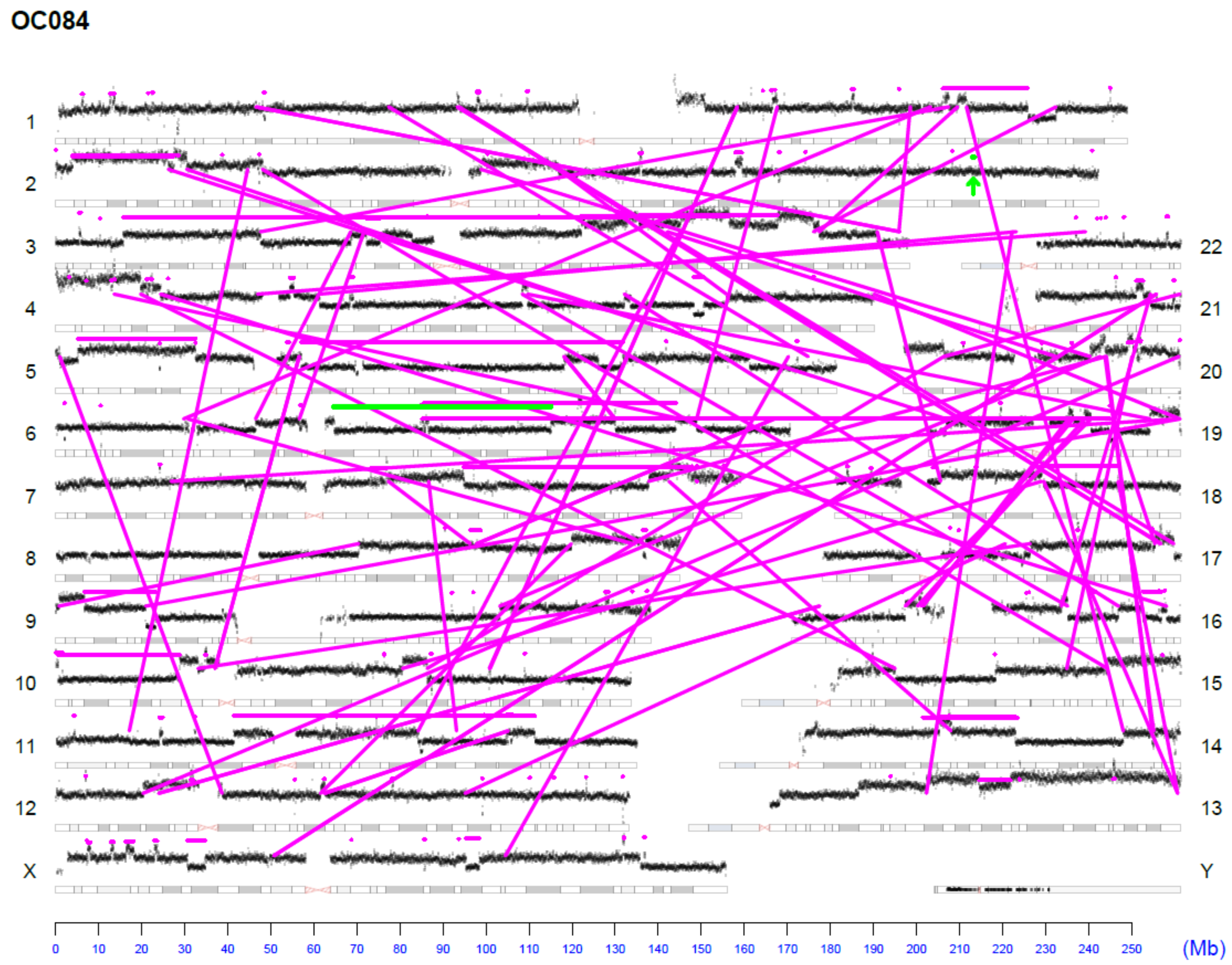

Supplemental Figure S8

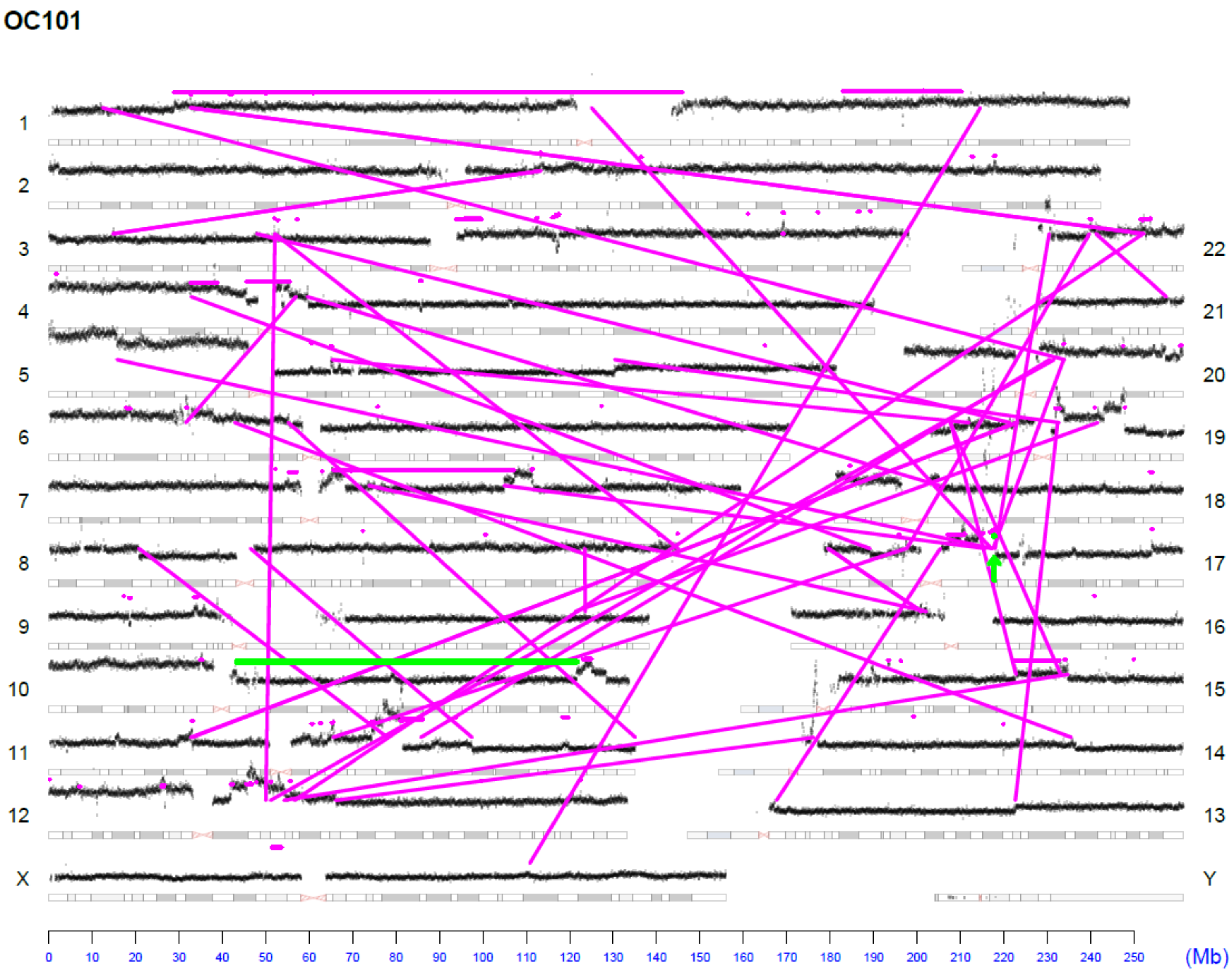

Supplemental Figure S1-8. Genome Plots. Plots for those not already in the main manuscript. Chromosomes are listed on the left and right Y-axis'; basepair position is on the X axis. Grey cytobands indicate genomic loci bands. The height of the black dots each represents the average number of reads over 30k bases. Magenta dots indicate small intrachromosomal rearrangements, while the magenta lines indicate interchromosomal rearrangements or larger intrachromosomal junctions. Green lines/dots indicate the junctions of interest monitored in the blood.

Supplemental Figure S9

A.

Case	Pre-surgical serum (ng/mL)	Pre-surgical plasma (ng/mL)	Post-surgical plasma (ng/mL)
OC004	NT	18	5.7
OC024	NT	23.3	14.7
OC049	22.7	35	9
OC058	NT	15	11.7
OC063	NT	13	17.3
OC067	20.7	12	32.3
OC068	NT	14.7	24
OC073	63.3	28.7	18.3
OC084	NT	21	10
OC101*	91.3	12	11.3

B.

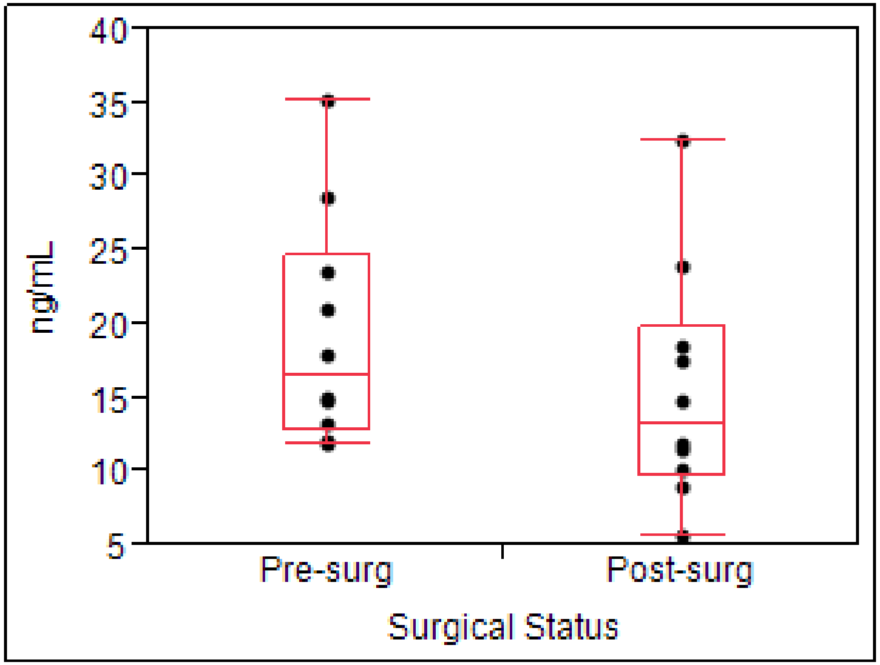

<u>Supplemental Figure S9</u>. **Total cfDNA.** A) Total yield of cfDNA obtained. *1.5mL used for OC101 pre-surgical serum. NT: not tested. B) Yields of cfDNA obtained in pre-surgical plasma compared to post-surgical plasma.

Supplemental Figure S10.

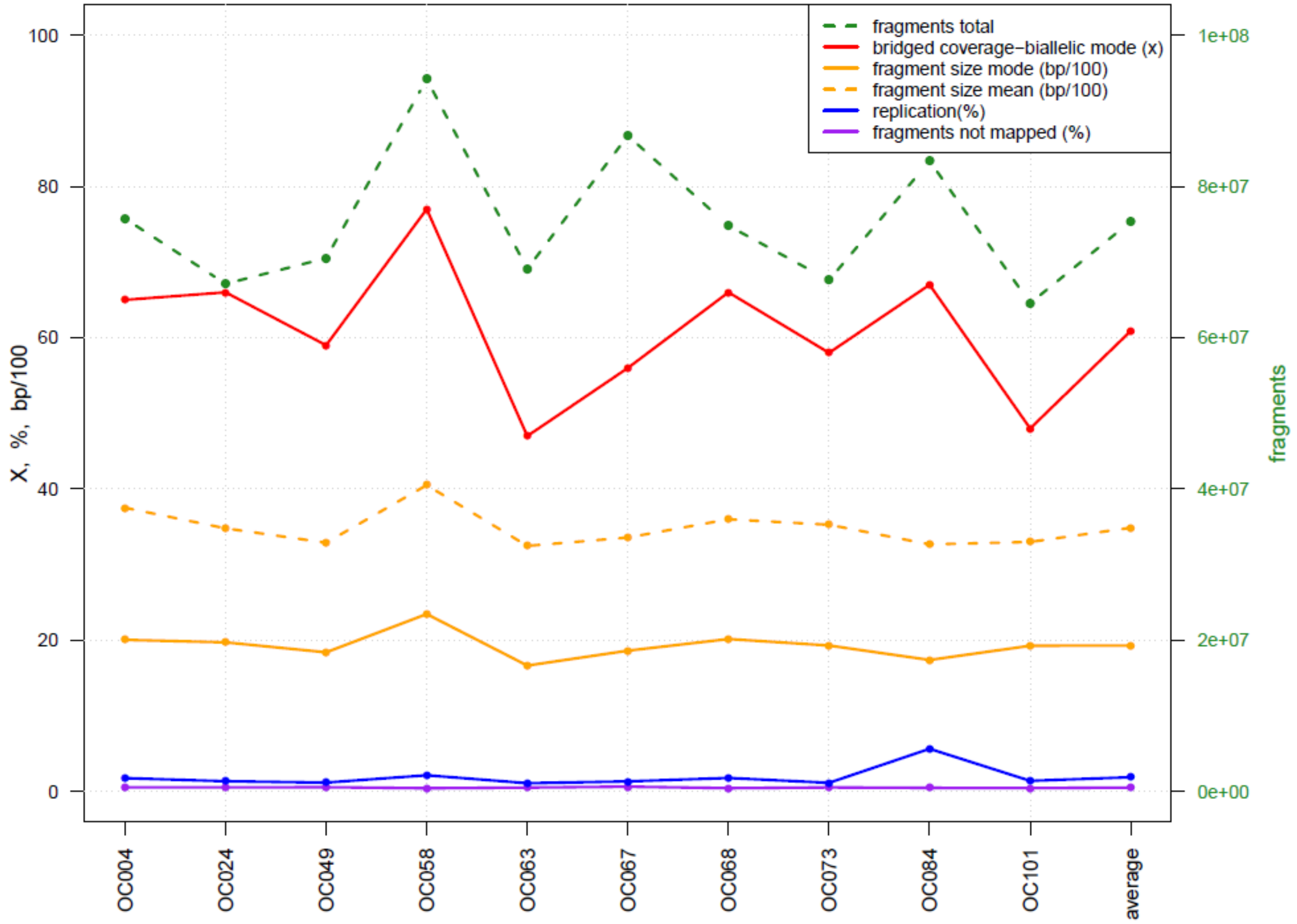

Supplemental Figure S10. Mate-pair sequencing coverage. The bi-allelic bridged coverage for these 10 samples averaged 61x, ranging from 47-77x. Bridged coverage, the estimated number of fragments (read-pairs) spanning a given position on the reference genome, is a function of the number of unique fragments sequenced for the sample and the length of each fragment. The samples had an average of 75 million total fragments (right vertical axis), with a low replication rate, averaging 2%. All replicate fragments are removed as the first step in the SVAtools analysis. The fragment length distribution for each sample reveals a skewed bell curve; resulting in a fragment length mean (mean length=3487 bp) that is 1556 bp larger than the peak of the distribution curve (mode length=1931 bp). Fragment lengths were divided by 100 to scale to the left vertical axis. Less than 1% of all fragments did not map to the reference Genome, GRCh38.

<u>Supplemental Table S1.</u> Description of junctions tested. Genome position and primer sequences for each rearrangement are indicated.

Case ID	Number of Supporting Mate Pairs	Chrom-osome A	Chrom-osome B	Position A of representative mate-pair (GRCh38)	Position B of representative mate-pair (GRCh38)	Locus A	Locus B	Gene A	Gene B	Event size of intra-chrom-osomal rearrangements (bp)	Putatively Targetable?	Primer A Sequence for qPCR	Primer B Sequence for qPCR	Product Size (bp)
OC004	8	2	2	214500173	214750815	2q35	2q35	VWC2L	BARD1	250000	yes	ggctaaaacaagatgtatcaggctagtc	GAGTGTTCAGTAAGTGTGTGCTGAATGC	170
OC004	20	4	4	102513691	102661946	4q24	4q24	NFKB1	MANBA	141000	yes	GATAGCTTGGGACCAGGAAGTCGAG	gccttttcaaattgcttctggatatacc	258
OC004	21	8	8	140868236	140969369	8q24.3	8q24.3	PTK2	PTK2	102000	yes	gacaaagggcacacctaatttgg	CACCTTTGAGCAGTGCCAGTCTAG	227
OC004	18	14	14	19468136	20360320	14q11.2	14q11.2	no_gene	PARP2	783000	yes	gatgttcctcacactgcgtatgc	GGAAGTGTGTTGTTTATCTGTGTTGTG	227
OC024	37	1	1	39424833	66009257	1p34.3	1p31.3	MACF1	PDE4B	26582000	no	GCCTTCCGTTTCCAGTACACTTGC	gtgatgccatactcagcctcatcatc	130
OC024	60	13	13	48272756	48309939	13q14.2	13q14.2	no_gene	RB1	42000	no	gcattctacccaccaaattctaatcag	GGTGGGAGGCATTTATGGAGG	144
OC049	17	1	1	155033892	156025421	1q22	1q22	DCST1	no_gene	989000	yes	GGGGAAATTGAGTCCAGAGAAGG	ctcaatagcaacaaaacaaaacaaacac	55
OC049	9	1	1	42956265	43140388	1p34.2	1p34.2	SLC2A1-AS	no_gene	176000	yes	GGATGGTGTGAAACACATAGGTAAGC	gagcagaatctcactatgttgtcccag	79
OC058	24	3	3	141255158	141349878	3q23	3q23	PXYLP1	ZBTB38	86000	no	CCAGGGTCCTATCTCCTCCCAAC	gaccagttgcttgcttcatacttatcc	115
* OC058	22	4	4	53422008	55407478	4q12	4q12	FIP1L1	TMEM165	1992000	no	gcagtcatggtaggcatagctttgc	GGCAGAATAATGAAGCCCGTTTGAC	99
OC063	8	1	1	153951573	154354284	1q21.3	1q21.3	CRTC2	ATP8B2	404000	yes	atctggggactgcccttcctc	ggacatgtgtgggttccaggac	65
OC063	9	3	3	78910459	79115587	3p12.3	3p12.3	ROBO1	ROBO1	205128	no	atggcttgtgctcactggaag	GCCAGTTAAAATGAGTGACTTCAAGTT	69
OC063	14	4	4	161938467	176795255	4q32.2	4q34.3	FSTL5	no_gene	14856788	no	gaaattttgtgacagaaatgaaaggac	AGGGAGGACAAGAACTCGGGA	67
OC063	3	17	17	43070545	43189953	17q21.31	17q21.31	BRCA1	NBR1	119408	yes	gtacctttctctcctgggtcctga	CTGTTTTTAGCAGGACTGTTTTTAGCTG	55
OC067	14	2	9	217530038	125630623	2q35	9q33.3	DIRC3	MAPKAP1	NA	yes	CCATCAAAGGGGTGCTATAAAATAGATC	tccctacttttgctaaccacagcc	80
OC067	11	17	17	74827720	75050587	17q25.1	17q25.1	TMEM104	KCTD2	222000	no	GTAAAACCTGGCTCACAGCAATCG	ctcaagaagcttgaagtcggcc	151
OC068	16	6	20	92413168	46245626	6q15	20q13.12	no_gene	CDH22	NA	no	GGATCACCTGAGGTTGGGAGTTC	gggccatctcagtctcttcacc	63
OC068	54	9	9	76625384	96299846	9q21.2	9q22.32	PRUNE2	HSD17B3	19827000	yes	ggttgtggaaacgctgtgaagc	gtcaggagttcgagaccagcc	65
OC068	24	19	19	55261376	56799006	19q13.42	19q13.43	HSPBP1	ZIM2	1539000	yes	ggactggagtactacttttcaacaatcag	caagatttattgcttcctgagttttcac	110
OC073	19	2	2	29567208	29683942	2p23.2	2p23.2	ALK	ALK	117000	no	GGGTTCCCAGTCTCTCTGTAATAAAGC	gggtatttctttcccatttcttgtcc	101
OC073	13	3	3	48188489	48261333	3p21.31	3p21.31	CDC25A	ZNF589	70000	yes	GGAGAAGTTGCTTACTGATTGGTGG	gagccaggatcacgccactg	95
OC073	4	11	11	13960361	35139867	11p15.2	11p13	no_gene	CD44	21184000	yes	cctctaagaaggatgtactggcaccc	CCGTAACTCCCACCTGAAGAGCAG	91
OC084	3	2	2	212873139	213482206	2q34	2q34	ERBB4	no_gene	609000	no	acatacaagggaaactccacaaagc	CATATTTAATTCATCTTCATGTCCTTGGTG	69
OC084	14	6	6	64735029	115063000	6q12	6q22.1	EYS	no_gene	49941000	no	tagcaaaacagcctaatcaaatatcattg	GACATGATTTAGTTTTAAGTCAGTCAACTC	140
OC101	8	10	10	43250167	121579008	10q11.21	10q26.13	RASGEF1A	FGFR2	79594000	yes	gattgatttactgcttctgctttgcc	CTTTCTGATGGCAGATGTGTAAGTGC	78
OC101	11	17	17	39548840	39717320	17q12	17q12	no_gene	ERBB2	174000	yes	gagaccagcctcatcgacctaata	caccccttccaggctcttttctg	77

* Both breakpoints for this junction were tested
